# Advances of curcumin in nervous system diseases: the effect of regulating oxidative stress and clinical studies

**DOI:** 10.3389/fphar.2024.1496661

**Published:** 2024-11-01

**Authors:** Yuxun Wei, Hong Li, Yue Li, Yue Zeng, Tian Quan, Yanen Leng, En Chang, Yingtao Bai, Yuan Bian, Yi Hou

**Affiliations:** ^1^ Pharmacy Department, Clinical Trial Institution, The People’s Hospital of Zhongjiang, Deyang, China; ^2^ West China School of Medicine, Sichuan University, Chengdu, China; ^3^ Molecular Urooncology, Department of Urology, Klinikum Rechts der Isar, Technical University of Munich, München, Germany; ^4^ Department of Oncology, 363 Hospital, Chengdu, China

**Keywords:** curcumin, neurological disorders, oxidative stress, antioxidant properties, molecular mechanism

## Abstract

In recent years, researchers have highly observed that neurological disorders (NSDs) with the aging of the population are a global health burden whose prevalence is increasing every year. Previous evidence suggested that the occurrence of neurological disorders is correlated with predisposing factors such as inflammation, aging, and injury. Particularly, the neuronal cells are susceptible to oxidative stress, leading to lesions caused by high oxygen-consuming properties. Oxidative stress (OS) is a state of peroxidation, which occurs as a result of the disruption of the balance between oxidizing and antioxidizing substances. The oxidative intermediates such as free radicals, hydrogen peroxide (H_2_O_2_), and superoxide anion (O^2^-) produced by OS promote disease progression. Curcumin, a natural diketone derived from turmeric, is a natural antioxidant with a wide range of neuroprotective, anti-inflammatory, anti-tumor, anti-aging, and antioxidant effects. Fortunately, curcumin is recognized for its potent antioxidant properties and is considered a promising candidate for the prevention and treatment of neurological diseases. Consequently, this review elucidates the mechanisms by which curcumin mitigates oxidative stress and emphasizes the potential in treating nervous system disorders, including depression, Alzheimer’s disease, Parkinson’s disease, epilepsy, subarachnoid hemorrhage, and glioblastoma. We aim to provide a new therapeutic option for the management of neurological diseases.

## Graphical Abstract

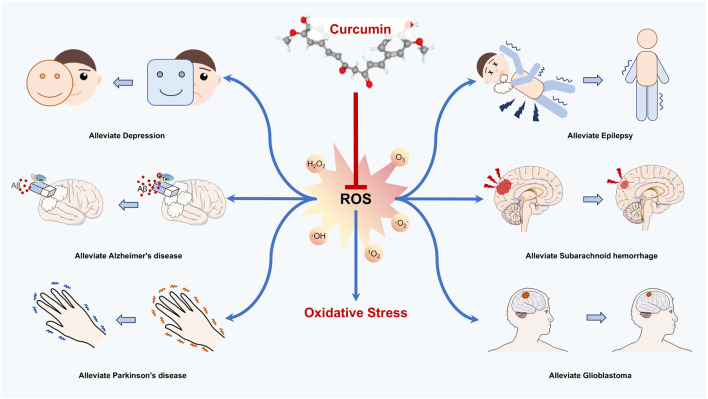

## Introduction

Neurological disorders are diseases characterized by neurological damage and functional impairments resulting from various etiologies. These disorders can affect individuals at any age and are particularly prevalent among the elderly. As the global population ages, the number of individuals suffering from neurological disorders is expected to rise, placing significant strain on both human health and economic resources (Birbeck et al., 2015). Clinical manifestations of neurological disorders primarily include neurodegenerative diseases, epilepsy, arachnoid hemorrhage, and glioma. The etiology of these disorders is complex, involving factors such as genetic inheritance, oxidative stress, infections, trauma, cerebrovascular lesions, and metabolic abnormalities (Valori et al., 2019; Nimgampalle et al., 2023; Tang et al., 2023). Currently, most neurological disorders, especially neurodegenerative diseases like Alzheimer’s and Parkinson’s are considered irreversible (Abdolmaleky and Zhou, 2023). For instance, the different forms of amyloid β-protein (Ab) linked with the decreased overproduction, aberrant aggregation, or elimination are key events in the pathogenesis of Alzheimer’s disease (AD) (Ferrari and Sorbi, 2021; Fang et al., 2019), and oxidative stress may contribute to the developmental process (Monteiro et al., 2023; Ullah et al., 2024). Meanwhile, the occurrence of depression involves complex molecular mechanisms related to dysfunction of the monoaminergic nervous system, abnormality of the hypothalamic pituitary adrenal axis, imbalance of calcium homeostasis and calcium signaling pathways, mitochondrial dysfunction, autophagy, cell apoptosis, and oxidative stress (Bansal and Kuhad, 2016; Villa et al., 2018; Bhatt et al., 2020). Moreover, the clinical therapy of neurological disorders tends to be largely symptomatic due to the intricate etiology, suffering from the disadvantages of side effects, drug resistance, and insensitivity (Nimgampalle et al., 2023). Antidepressants commonly used in clinic, such as tricyclic antidepressants, monoamine oxidase inhibitors, selective 5-HT reuptake inhibitors, and 5-HT-norepinephrine reuptake inhibitors, have side effects as dry mouth, blurred vision, inability to drive, sexual dysfunction, headache, gastrointestinal symptoms, anxiety, and agitation (Barkin and Fawcett, 2000; Feighner, 1999; Fava, 2000). Unfortunately, the most existing therapy modalities for PD only target symptoms. Dopamine supplements temporarily control motor dysfunction caused by degeneration of the dopaminergic nigrostriatal system. Meanwhile, the drugs in PD including levodopa, dopamine agonists, COMT inhibitors, MAO-B inhibitors, anticholinergics, and amantadine still have unavoidable side effects as motor complications, hallucinations, insomnia, gastrointestinal disturbances, cognitive deficits, dry mouth, and constipation (Khanam and Siddique, 2018; Kwon et al., 2022; Faulkner, 2014). Consequently, research into the pathogenesis of various neurological disorders and more effective pharmacological interventions has become a major focus within the field of neurology.

Redox reactions constitute the primary chemical basis for energy conversion and metabolism in humans. Unfortunately, oxidative stress resulting from an imbalance between oxidative and antioxidant effects could lead to a variety of negative outcomes (Jones, 2024). Free radicals and reactive oxygen species (ROS), including superoxide anions (O_2_^−^), hydrogen peroxide (H_2_O_2_), and hydroxyl radicals (·OH) are the principal contributors to oxidative stress in the body. These species are correlated with electron transfer and the generation of various active intermediates through chemical reactions. Mitochondria, as crucial organelles for ATP production, are significant producers of reactive oxygen species. Fortunately, various reductases such as superoxide dismutase (SOD), catalase (CAT), glutathione peroxidase (GSH-Px), along with reducing compounds-glutathione, vitamin C, and vitamin E, help mitigate the harmful effects of oxidative stress *in vivo* (Sies, 2021). The nervous system is particularly vulnerable to oxidative stress due to the high oxygen consumption and susceptibility to depletion (Jones, 2015; Tonnies and Trushina, 2017; Maiese, 2023). In recent years, the impact of oxidative stress on various neurological disorders has been the focus of extensive research. Notably, oxidative stress in the context of neurodegenerative diseases has been shown to induce neuronal cell death and abnormal protein aggregation (Birla et al., 2020), which are key predisposing factors. However, the development of drugs targeting oxidative stress remains a challenge at this stage.

Curcumin (CU), a diketone compound derived from turmeric, has been widely utilized globally as a natural pigment, which has demonstrated various pharmacological effects, including anti-inflammatory, immunomodulatory, antioxidant, and anti-tumor properties (Kocaadam and Sanlier, 2017; Zia et al., 2021). Notably, the diketone structure of CU, characterized by its excellent reducing properties, serves as a primary basis for its antioxidant action (Nelson et al., 2017). Recent studies indicate that CU inhibits neuronal damage caused by oxidative stress and effectively mitigates disease progression by eliminating free radicals and ROS while enhancing reductase activity (Samarghandian et al., 2017; Sarawi et al., 2021). Furthermore, the beneficial effects of CU on oxidative stress have been corroborated by clinical trials. This study reviews the pharmacological mechanisms through which CU acts as an antioxidant, particularly in alleviating the detrimental effects of oxidative stress on neurodegenerative diseases, which searched for relevant studies on ‘curcumin, oxidative stress, nervous system diseases, depression, Alzheimer’s disease, Parkinson’s disease, epilepsy, subarachnoid hemorrhage, and glioma’ by PubMed, Web of Science, and CNKI databases. Additionally, the findings from clinical trials involving CU in neurodegenerative diseases are discussed to provide a foundation for future research on the antioxidant effects of CU in this context.

## Physicochemical properties and metabolism

**FIGURE 1:**
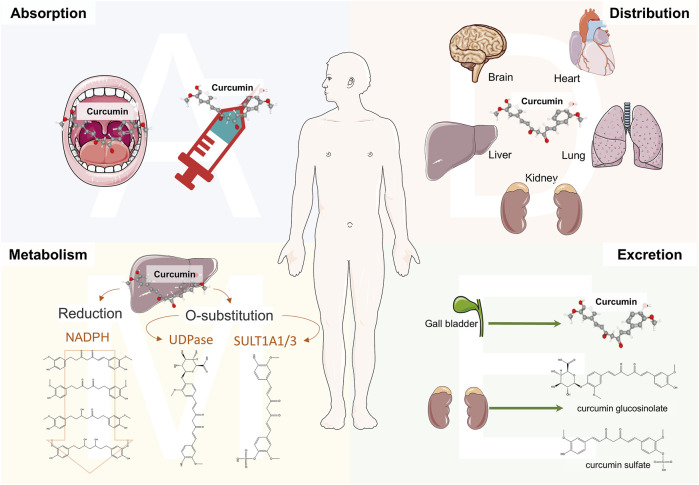
Schematic diagram of the pharmacokinetics of curcumin *in vivo*. The administration methods of curcumin (CU) include oral and the injection of the vein and abdominal cavity. CU entering the body is mainly distributed in the liver, kidneys, brain, and lungs. Particularly, CU could cross the blood-brain barrier in the brain, which is the basis for treating neurological disorders. The liver is the main metabolizing organ for curcumin and can generate a range of metabolites such as CU glucosinolates, CU sulfates, and hydrogenated curcuminoids by O-substitution and reduction pathways. Renal excretion is the metabolic pathway for CU glucosinolate and CU sulfate. Meanwhile, CU can be excreted through bile.

Curcumin, with the molecular formula C_21_H_20_O_6_, exhibits a polyphenolic chemical structure. The lipid-water partition coefficient of CU is LogP: 3.2, indicating extremely low solubility in water, while it is soluble in organic solvents such as DMSO, methanol, and acetone (Payton et al., 2007). Notably, CU possesses enolization properties due to its β-dicarbonyl structural unit, which contributes to its instability and irregular metabolism. However, the 1,3-diketone structure is also a crucial motif for protecting nerve cells from oxidative stress (LoPachin et al., 2011). In acidic or neutral solvents, CU primarily exists in its ketone form, while in alkaline solvents, acidic structures predominate, enhancing stability (Priyadarsini, 2014). Interestingly, the electron cloud conjugation effect of CU increases with solvent basicity, leading to pronounced deprotonation, which enhances solubility but also increases susceptibility to degradation (Ghosh et al., 2023; Pan et al., 2014). Consequently, due to these properties such as solubility and stability in various solvents, CU exhibits low bioavailability in the human body (Figure 1), necessitating a more complex mode of administration. A peak serum concentration of 0.22 μg/mL was observed 1 h after oral administration at a dose of 1.0 g/kg in a mouse model, while a peak concentration of 2.25 μg/mL was recorded 0.25 h after intraperitoneal administration at the same dose (Pan et al., 1999). Additionally, the area under the curve (AUC) for CU in rats was found to be 3.6 ± 0.6 min/μg/mL following oral administration of 500 mg/kg, and only 7.2 ± 1.2 min/μg/mL after intravenous administration of 10 mg/kg, further indicating poor absorption characteristics (Yang et al., 2007). Studies utilizing radioactive elements to investigate the distribution of CU have identified its presence in the liver, heart, kidneys, brain, lungs, and muscles of mice administered intraperitoneally (Perkins et al., 2002). Notably, Pan et al. (1999) demonstrated that CU can penetrate the brain, albeit at a concentration of only 0.4 ± 0.01 μg/g. These findings further substantiate the notion that CU is capable of crossing the blood-brain barrier, which is critical in the context of various neurological disorders, thereby exerting pharmacological effects. The liver serves as the primary organ for CU metabolism, which occurs through two principal pathways: O-substitution and reduction. The phenolic hydroxyl group of CU interacts with glucuronic acid and sulfate, resulting in the formation of CU glucosinolate and CU sulfate, respectively. The human phenol sulfotransferase isozymes SULT1A1 and SULT1A3 play essential roles in the sulfate substitution of CU (Ireson et al., 2002). Concurrently, the UDP enzyme family, a group of glucuronosyltransferases, facilitates the glucuronosyl substitution of CU in the human liver and gastrointestinal tract (Hoehle et al., 2007). The NADPH enzyme is responsible for reducing CU to dihydrocurcumin, which can further convert to tetrahydrocurcumin within the reduction pathway (Wang et al., 2021). Interestingly, these reduction products can undergo subsequent reduction reactions, yielding hexahydrocurcumin, octahydrocurcumin, and various ferulic acid analogs (Ireson et al., 2001). The elimination of CU is closely associated with the route of administration, as the majority of orally administered CU is excreted in feces. A clinical trial investigating long-term oral administration revealed that CU was present in the feces of subjects, while it was not detected in urine (Sharma et al., 2001). Furthermore, the studies revealed the presence of CU glucosinolates and sulfates in the urine of rats; however, the parent compound was not detected in the urine (Ravindranath and Chandrasekhara, 1980). A clinical study also demonstrated that CU was virtually undetectable in urine, with only the glucuronide conjugate observed (Irving et al., 2013). Notably, rats administered CU via intraperitoneal or intravenous routes exhibited biliary excretion (Holder et al., 1978; Lee et al., 2012). These physicochemical properties and the pharmacokinetic profile of CU *in vivo* indicate low bioavailability. Consequently, researchers have developed new formulations aimed at improving the half-life and bioavailability of CU.

## Depression

Depression is a prevalent mental disorder characterized by a persistent low mood and associated mental symptoms, including self-harm and suicidal ideation (Martino et al., 2022; Boumparis et al., 2016; McCarron et al., 2021). Currently, researchers have identified several factors that may contribute to the etiology of depression, including biochemical, neuroendocrine, neuroimmunological, genetic, and psychosocial influences (Menard et al., 2016; Penner-Goeke and Binder, 2019; Idunkova et al., 2023). However, the specific pathogenesis of the disorder remains unclear. Oxidative stress has long been recognized as a significant factor influencing depression. The phosphorylation of ATP within cells generates reactive species, including ROS, reactive nitrogen species, and free radicals (Brookes et al., 2004; Strzyz, 2022; Bando et al., 2021; Leite-Aguiar et al., 2024). While ROS plays a crucial role in the growth and development of nerve cells under physiological conditions, an excess of ROS can disrupt the balance of antioxidants, leading to oxidative stress, which adversely affects normal physiological functions. Glutathione (GSH) is an important antioxidant that scavenges ROS and free radicals, thereby preventing cellular peroxidation (Liu et al., 2022). The oxidized form of GSH can be regenerated into its reduced form by the action of reductases and NADPH (Zeeshan et al., 2016). Additionally, superoxide dismutase (SOD) catalyzes the conversion of superoxide radicals into hydrogen peroxide (H_2_O_2_), which is subsequently degraded by catalase. Recent studies have increasingly focused on the roles of GSH and SOD in the context of depression (Gibson et al., 2012; Camkurt et al., 2016). The brain, being the most oxygen-demanding organ, is particularly vulnerable to oxidative stress due to its high content of nerve cells and the abundance of neurofunctional substances such as lipids and proteins that are prone to oxidative damage (Hulbert et al., 2007). The peroxidation of these substances results in oxidation products that contribute to physiological processes, including decreased cellular function and cell death. Research indicates that levels of malondialdehyde (MDA) in the brains of patients with depression are higher than those in healthy individuals; notably, MDA is produced through lipid oxidation in nerve cells (Maes et al., 2011; Almulla et al., 2023). Nitric oxide (NO), a product of L-proline oxidation, is also found at elevated levels in depressed patients (Salim, 2017; Piper et al., 2023). Further studies have demonstrated that the inhibition of NO production can mimic the effects of antidepressants (Ghasemi, 2019). Additionally, the inhibitory effect of peroxides on telomerase represents a significant cause of cellular DNA damage. The 8-hydroxy-2′-deoxyguanosine (8-OHdG) is a modification product resulting from oxidative DNA damage induced by ROS. Research has shown that individuals with depression, particularly those experiencing recurrent episodes, exhibit significantly elevated levels of 8-OHdG compared to normal levels (Omari Shekaftik and Nasirzadeh, 2021; Syafrita et al., 2020). This finding suggests a strong positive correlation between oxidative stress and DNA damage in depressed patients.

Curcumin, a natural antioxidant, influences various oxidative reactions within the body. Previous studies have indicated that depression is often associated with decreased levels of antioxidants. The current study demonstrated that the depressive behavior of rats infected with Toxoplasma gondii improved following the administration of CU (Moradi et al., 2023). Additionally, this research revealed an increased expression of GSH and SOD enzymes in the hippocampus of the rat brain, along with a reduction in MDA levels, thereby confirming that CU effectively mitigates the impact of oxidative stress on depression. Nrf2, a key endogenous antioxidant transcription factor, is activated by the dissociation of the Keap1 protein, subsequently entering the nucleus to regulate the expression of antioxidant genes in conjunction with ARE elements (Vriend and Reiter, 2015). Furthermore, studies have identified Nrf2 as a promising antioxidant target for depression treatment (Dang et al., 2022). Continuous administration of CU at a dosage of 100 mg/kg/day for 28 days in CUMS model rats significantly alleviated depressive behaviors, including those observed in the SPT, forced swimming, and novelty inhibition tests (Liao et al., 2020). Further mechanistic studies have demonstrated that CU intake in rats leads to an increase in oxidative stress markers, including 8-OHdG, Nox2, and 4-HNE. Notably, prolonged CU intake reversed the inhibition of Nrf2 protein and activated the expression of the antioxidant enzymes NQO-1 and HO-1 via the Nrf2-AEB pathway in a depressed model of animals. A lipopolysaccharide-induced model of moderate depression (MDD) in rats further illustrated that CU enhances antioxidant activity by elevating Nrf2 expression (Fan et al., 2021). Additionally, this study confirmed that CU reduced the rise in miR-146a-5p levels and reversed the decrease in the expression of the p-ERK signaling pathway in glial cells, thereby protecting synaptic neurons in the CA1 region of the hippocampus. To address the low activity associated with curcumin’s low bioavailability, curcumin was formulated into nanocapsules, which proved more effective in enhancing antioxidant substances, such as the SOD enzyme, in Aβ25-35 protein-induced model mice (Fidelis et al., 2019). Furthermore, curcumin-zinc oxide nanoparticles significantly increased GSH concentrations in the cerebral cortex, hippocampus, and striatum compared to curcumin alone, and more effectively reduced MDA levels (Fahmy et al., 2024).

Multiple clinical trials have demonstrated the positive effects of CU on depression (Table 1). Notably, CU exhibits significant efficacy in major depressive disorder (MDD), whether administered alone or in combination with other antidepressants or monomers (Lopresti and Drummond, 2017; Sanmukhani et al., 2014). Combined CU extract plus saffron (15 mg bid) in a randomized, double-blind, placebo-controlled studyfor 12 weeks was associated with significantly greater improvements in depressive symptoms compared with placebo (*p* = 0.031), which the Spielberger State-Trait Anxiety Inventory state (STAI-S) scores (*p* < 0.001) and STAI-trait (STAI-T) scores (*p* = 0.001) decreased significantly. A 6-week clinical trial using fluoxetine as a positive control found that oral administration of CU at a dose of 1,000 mg/day produced effects comparable to those of 20 mg/day fluoxetine, with response rates on the 17-item Hamilton Depression Rating Scale (HAM-D17) exceeding 60% for both treatments, significantly outperforming placebo (12.5%–51.8%) (Sanmukhani et al., 2014). Interestingly, when CU was combined with fluoxetine, the HAM-D17 score of the subjects reached 77.8%, although this result was not statistically different from the effects of CU or fluoxetine alone. Additionally, CU has been shown to positively impact depression associated with other medical conditions. For instance, a study indicated that patients with diabetes and peripheral neuropathy who ingested 80 mg/day of Nano-curcumin for 8 weeks exhibited improved depression and anxiety scores compared to those receiving a placebo (Asadi et al., 2020). Furthermore, a dosage of 1 g/day of CU led to improvements in depression and behaviors associated with coke oven exposure in obese patients, as evidenced by a significant decrease in their Beck Anxiety Inventory (BAI) scores (Esmaily et al., 2015). Notably, CU also alleviates depression and anxiety-related symptoms in healthy menopausal women, with observed decreases in MDA levels and improvements in antioxidant capacity (Farshbaf-Khalili et al., 2022). This study further supports the notion that CU alleviates depression in humans by inhibiting oxidative stress.

**TABLE 1:** Clinical research of CU in the treatment of depression.

Treatment group	Control group	Sample size (treatment/control)	Treatment time (weeks)	Results	Reference
Low-dose Curcumin extract (250 mg, bid), high-dose curcumin extract (500 mg, bid), combined low-dose curcumin extract plus saffron (15 mg, bid)	Placebo	26, 30, 24/31	12	Improvements in depressive symptoms, STAI status, and STAI trait scores, especially in patients with atypical depression	Lopresti and Drummond (2017)
Fluoxetine (20 mg/d), curcumin (1,000 mg/d), fluoxetine (20 mg/d) + curcumin (1,000 mg/d)	Placebo	17, 16/18	6	Mean changes in HAM-D17 scores were comparable	Sanmukhani et al. (2014)
Nano‐curcumin (80 mg/d)	Placebo	35/37	8	The mean scores for depression and anxiety were both reduced	Asadi et al. (2020)
Curcumin (1 g/d, 4 g/d)	Placebo	15/15	4	Decrease in Beck Anxiety Inventory (BAI) score	Esmaily et al. (2015)
Curcumin (500 mg, bid)	Placebo	26/28	8	Decrease in MDA, hs-CRP, TAC, etc.	Farshbaf-Khalili et al. (2022)

## Alzheimer’s disease

**FIGURE 2:**
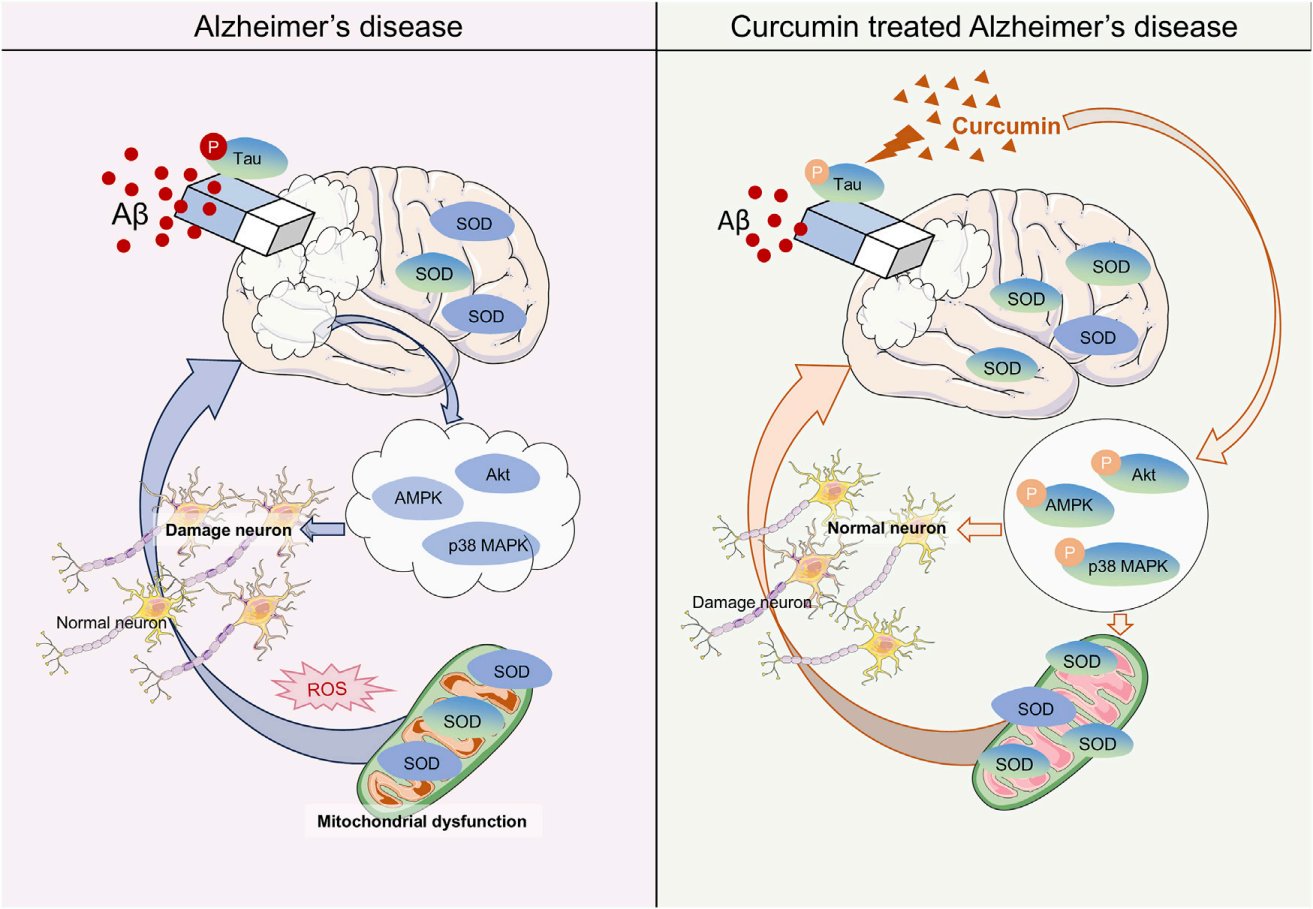
Mechanisms of Curcumin in the Treatment of Alzheimer’s Disease (AD). The mechanism of AD by affecting oxidative stress with Curcumin (CU). Curcumin reduces the AB and the hyperphosphorylation of tau proteins in the brain. In neuronal cells, curcumin increases the activity of the SOD enzyme to reduce mitochondrial dysfunction caused by oxidative stress. Meanwhile, CU improved the phosphorylation process of the AKT/p38 MAPK pathway to reverse neuronal apoptosis caused by oxidative stress.

Alzheimer’s disease (AD) is a prevalent neurodegenerative disorder that leads to significant cognitive impairment, particularly in the elderly (Scheltens et al., 2021). The etiology of AD remains poorly understood (Rostagno, 2022). Generally, the primary pathological features of AD include the accumulation of Aβ protein, hyperphosphorylation of tau protein, and the formation of neurofibrillary tangles (Ferrari and Sorbi, 2021). The brain is an organ that is highly susceptible to oxygen consumption in humans, utilizing approximately 20% of the body’s oxygen (Ballance et al., 2019). Notably, neurons in the brain contain a substantial amount of lipids, nucleic acids, and proteins, all of which are particularly vulnerable to oxidation by reactive compounds in the body, leading to functional disorders in cells. Due to these characteristics, neurons are more susceptible to oxidative stress, which may induce a range of nervous system diseases, especially in the context of AD (Pratico, 2008) (Figure 2). Research has shown that oxidative stress can lead to an increase in phosphorylated tau protein through the P38/JNK pathway (Su et al., 2010). Additionally, oxidative stress promotes the cross-linking of tau protein with dityrosine (DIY), resulting in the formation of copolymers (Maina et al., 2021). These findings underscore the connection between oxidative stress and tau protein in the pathogenesis of AD. Previous studies have also reported that oxidative stress facilitates the deposition of amyloid beta (Aβ) (Andorn and Kalaria, 2000; Pratico et al., 2002). The Aβ peptide generates reactive oxygen species in the presence of transition metals such as copper and iron. Furthermore, similar to the tau protein, Aβ can form stable dityrosine cross-linked dimers with DIY under oxidative stress conditions, which exacerbates the detrimental effects of oxidative stress on neuronal cells (Smith et al., 2007). Most clinical trials have demonstrated that levels of oxidative coenzyme Q (an early marker of oxidative stress), 8-hydroxy-20-deoxyguanosine (a marker of DNA oxidative damage), and malondialdehyde (MDA, a lipid oxidation product) in the brains and blood of AD patients are significantly elevated compared to normal individuals (Isobe et al., 2010; Park et al., 2021; Nie et al., 2024). Mitochondria, which are key organelles involved in energy metabolism, facilitate the transfer of electrons through redox reactions to generate ATP. However, various mitochondrial dysfunctions can lead to abnormal electron transfer and reduced reductase activity, resulting in the production of harmful substances such as oxygen free radicals (Song et al., 2021; Bai et al., 2022). Notably, abnormal expression of oxidoreductase systems has also been documented in related studies (Nie et al., 2024). These foundational experiments and clinical investigations indicate that oxidative stress may influence the progression of AD through mechanisms such as mitochondrial dysfunction, alterations in the expression of catalytic enzyme systems, and DNA damage, thereby reinforcing the hypothesis of oxidative stress as a potential mechanism underlying AD.

Numerous studies have reported on the antioxidant properties of CU in the context of Alzheimer’s disease (AD). CU significantly improved cognitive impairment and memory deficits in AD model rats, with superoxide dismutase (SOD) levels in the brains of curcumin-administered rats being significantly higher than those in the AD model, in a dose-dependent manner. This research also validated the synergistic effect of curcumin with coenzyme Q10 in AD, further demonstrating curcumin’s antagonistic effect on oxidative stress (Kumar et al., 2023). Previous studies have shown that CU can reduce Aβ-induced oxidase activity and DNA damage. Furthermore, whether used for prevention, in combination, or as a treatment, CU can mitigate mitochondrial damage and cell apoptosis caused by Aβ-mediated oxidative stress, with this effect being most pronounced when CU is applied prophylactically. Interestingly, this influence in AD is closely related to curcumin’s ability to reverse the dephosphorylation of the AKT/p38 MAPK pathway induced by Aβ, thereby affecting downstream apoptotic proteins (Fan et al., 2017). Another study illustrated that CU restores Aβ-induced mitochondrial dysfunction and synaptotoxicity, further demonstrating that prophylactic application of CU represents the optimal stage for pharmacological intervention (Reddy et al., 2016). Meanwhile, research has shown that CU can reduce Aβ levels in Parkinson’s disease (PD) models and restore the number of damaged neurons, which is linked to increased superoxide dismutase (SOD) enzyme activity, reduced intracellular inflammation, and enhanced AMPK phosphorylation (Shao et al., 2023). However, the interplay of these three mechanisms in the context of PD remains unreported. SIRT1, a NAD+-dependent histone deacetylase, is known for up-regulating antioxidant and anti-inflammatory proteins (Liu et al., 2021). Bisdemethoxycurcumin (BDMC), a CU analog that retains the 1,3 diketone moiety, has been shown to protect neurons from oxidative stress damage by activating SIRT1 expression, suggesting its potential as a therapeutic compound for AD (Xu et al., 2020). Notably, a clinical trial involving 12 weeks of oral CU administration observed a reduction in insulin resistance in type 2 diabetes, which was associated with its effects on Aβ protein accumulation and tau protein hyperphosphorylation (Thota et al., 2020) (Table 2). This finding indirectly supports the therapeutic role of CU in AD. However, clinical trials assessing curcumin’s efficacy in AD have indicated that it does not appear to have a significant positive effect. In a randomized double-blind trial of oral CU over 24 weeks, no significant differences were observed in biomarkers or related psychiatric scores compared to the placebo (Ringman et al., 2012). Additionally, another clinical study reported similar findings (Baum et al., 2008). Importantly, both studies noted issues of low bioavailability, limited sample sizes, and short durations of CU administration. Therefore, further clinical trials are warranted to verify the effectiveness of CU in treating AD.

**TABLE 2:** Clinical research of CU in the treatment of Alzheimer’s disease.

Treatment group	Control group	Sample size (treatment/control)	Treatment time (weeks)	Results	Reference
Curcumin (180 mg/d)	Placebo	14/15	12	Decrease in GSK-3β and IAPP	Thota et al. (2020)
Curcumin C3 Complex^®^ (2 g/d, or 4 g/d)	Placebo	9, 10/11	24	The efficacy is not yet clear	Ringman et al. (2012)
Curcumin (1 g/d, 4 g/d)	Placebo	8, 11/8	48	Decrease in MiniMental State Examination (MMSE) scores	Baum et al. (2008)

## Parkinson’s disease

Parkinson’s disease (PD), similar to AD, is a prevalent neurodegenerative condition that profoundly affects individuals aged over 60, particularly those over 80 (Cacabelos, 2017; Hou et al., 2019; Alzheimer’s Association Report, 2020). The pathological features of PD include abnormal dopamine function in the substantia nigra of the brain and the presence of Lewy bodies formed by the aggregation of α-synuclein (α-syn) (Atik et al., 2016; Fayyad et al., 2019; Braak et al., 2003; Koeglsperger et al., 2023). The etiology of PD is generally believed to be closely related to environmental and genetic factors, as well as dysfunctions within the nervous system (Samii et al., 2004). Aging is considered one of the most significant contributors to the onset of PD (Collier et al., 2017). In recent years, oxidative stress has emerged as a predisposing factor for various neurological diseases (Imbriani et al., 2022). Dopamine neurons (DAns) in the substantia nigra pars compacta (SNPc) serve as key sites for the synthesis and storage of dopamine (DA) in the body, regulating human cognition and behavioral activity through the nigrostriatal pathway, midbrain limbic cortex system, nodular funnel system, and hypothalamic spinal tract. Notably, DA, as a neurotransmitter containing catecholamines, is not only easily oxidized but also generates free radicals and toxic metabolites, including ROS, dopaquinone (DAQ), and 3,4-dihydroxyphenylacetaldehyde (DOPAL) (Vaarmann et al., 2010; Nagatsu et al., 2022; Jinsmaa et al., 2020). Dopamine (DA) enhances sensitivity to ROS in their presence, exemplifying a typical positive feedback regulation. The adduct formed by the binding of dopamine quinone (DAQ) to sulfhydryl groups in cysteine residues exacerbates protein misfolding and functional loss (Kishida et al., 2021; Boo, 2022). Concurrently, 3,4-dihydroxyphenylacetaldehyde (DOPAL) can induce protein cross-linking and aggregation, further contributing to neurodegeneration (Jinsmaa et al., 2020). Mitochondria function as the cellular “energy suppliers,” producing ATP through oxidative phosphorylation. Under normal circumstances, O_2_ is reduced to H_2_O within the mitochondria. However, the electrons involved in mitochondrial shuttling can be easily transferred to the mitochondrial matrix, resulting in the conversion of O_2_ to superoxide (O_2_^−^), a critical source of mitochondrial oxidative stress (Boo, 2022; Drose and Brandt, 2008). The superoxide dismutase (SOD) enzyme in mitochondria converts O_2_^−^ into hydrogen peroxide (H_2_O_2_), which is then further processed by glutathione peroxidase (GPx) and peroxidase (PRx) to generate water (H_2_O), thereby facilitating the clearance of ROS (Ruszkiewicz and Albrecht, 2015; Averill-Bates, 2023). Unfortunately, mitochondrial dysfunction impedes this redox clearance mechanism, potentially involving mitochondrial electrons and calcium ions (Ca^2+^) in the formation of ROS, ultimately leading to neuronal dysfunction and apoptosis (Schmidt, 2012; Olson et al., 2005). These conditions may be linked to mitochondrial gene mutations, such as those in the Parkin, PINK1, and DJ-1 genes, which are associated with the development of PD, as well as exposure to toxic substances that can damage mitochondria, including heavy metals and 1-methyl-4-phenyl-1,2,5,6-tetrahydropyridine (MPTP) (Dionisio et al., 2021; Hao et al., 2010; van der Merwe et al., 2015).

**FIGURE 3:**
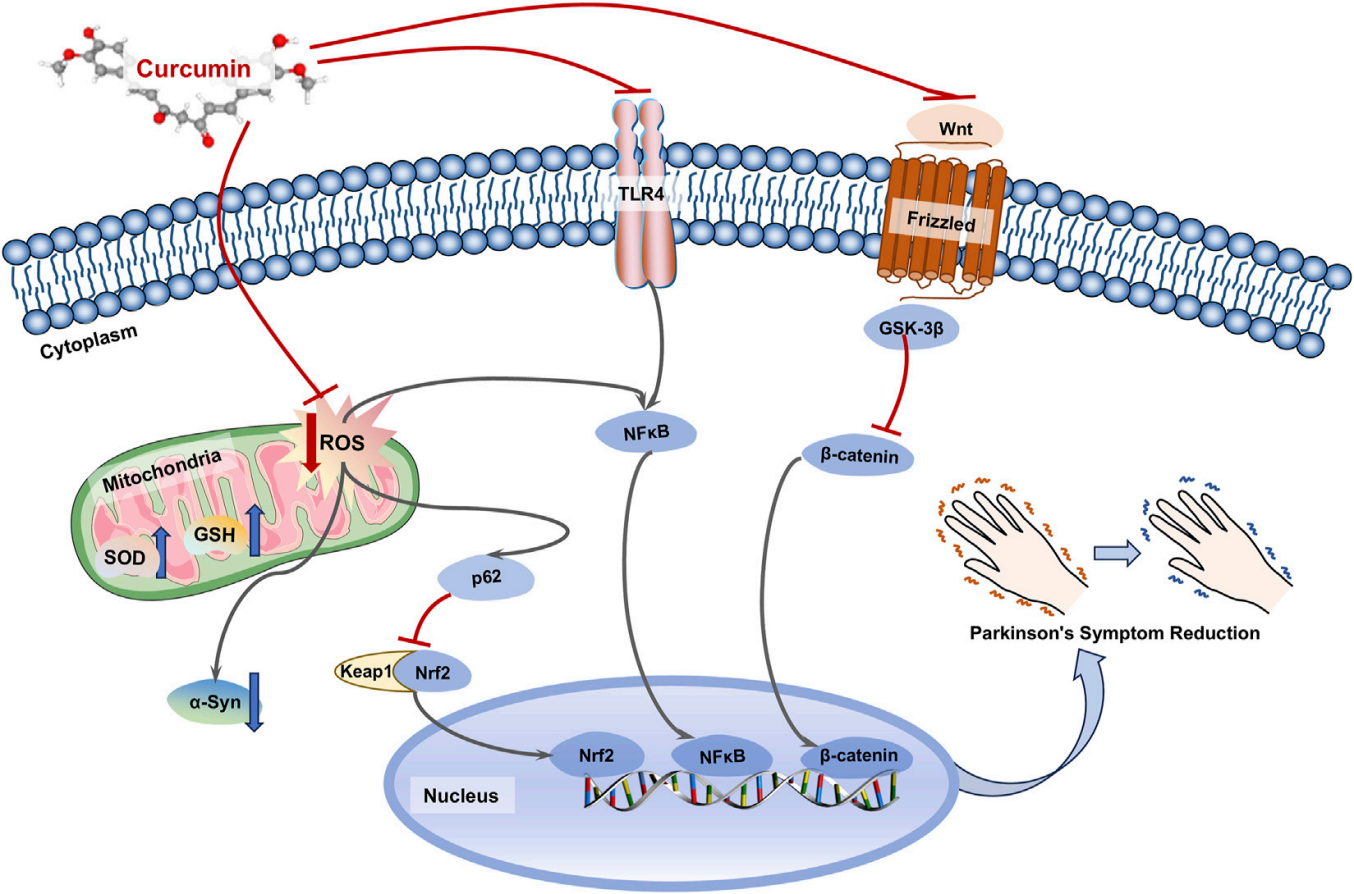
Mechanisms of curcumin in the treatment of Parkinson’s disease (PD). Curcumin (CU) could inhibit mitochondrial damage by oxidative stress in neuronal cells, and promote degradation of α-syn by autophagy through agonism of the Nrf2 transcription factor. Meanwhile, CU could inhibit inflammatory responses and intracellular antioxidant levels by affecting TLP4/NF-κB and Wnt/β-catenin pathways.

Due to its notable antioxidant properties, CU plays a significant positive role in PD (Figure 3). In a rotenone-induced PD model, both CU alone and in combination with levodopa or rasagiline improved motor disorders and behavioral impairments in the model mice by reducing oxidative stress and DNA damage (El-Shamarka et al., 2023). Moreover, CU improved the histopathological changes induced by rotenone, and CU showed additive effects on L-dopa or rasagiline with neuroprotective. Additionally, CU upregulated the expression of Nrf2 in the p62-Keap1-Nrf2 pathway, which is an antioxidant and autophagic transcription factor, thus reversing mitochondrial and oxidative stress damage in the rotenone-induced PD model (Rathore et al., 2023). Furthermore, this research demonstrated that CU could degrade the levels of α-syn by enhancing the autophagic function of nerve cells, suggesting that CU may mitigate oxidative stress-induced PD through multiple pathways. Oxidative stress is closely associated with inflammatory responses. NF-κB serves as a crucial nuclear transcription factor *in vivo*, and its activated form can translocate to the nucleus to regulate the expression of various genes related to inflammation, apoptosis, and immunity. Studies have indicated that oxidative stress can influence the expression of NF-κB and exacerbate neuronal inflammation in PD (Sivandzade et al., 2019). CU has been shown to reverse the inflammatory response induced by MPP+ in brain astrocytes by inhibiting the expression of pro-inflammatory effectors such as TLR4 and NF-κB, while simultaneously increasing GSH levels to reduce ROS. This suggests that CU may exert therapeutic effects on PD through its anti-inflammatory and antioxidant properties in neuronal cells (Yu et al., 2016). A clinical trial investigating CU supplementation for therapeutic purposes revealed that patients receiving CU experienced a decrease in scores on the COMPASS-31 autonomic and the non-motor symptom (NMSS) questionnaires, as well as a reduction in phosphorylated α-synuclein (p-syn) levels in cutaneous nerves (Donadio et al., 2022). Notably, CU levels were monitored in the blood and cerebrospinal fluid of treated patients, supporting its potential therapeutic effects on central nervous system disorders. Furthermore, the Wnt/β-catenin signaling pathway has been confirmed to be associated with the development of AD (Wang et al., 2022; Ghasemi et al., 2023). Additionally, another study indicated that CU activates the Wnt/β-catenin signaling pathway by inhibiting the expression of GSK-3β, which may be a key protein through which CU influences this pathway in PD (Zhang et al., 2011).

## Epilepsy

Epilepsy, the most prevalent disorder of the central nervous system (CNS), affects over 50 million individuals globally, posing a significant threat to human health (Thijs et al., 2019). This condition is characterized by recurrent unprovoked seizures, which are generally classified as either generalized or partial. Contemporary research indicates that potential causes of epilepsy include stroke, traumatic brain injury, and neurological infections. Pathologically, epilepsy is characterized by abnormal discharges of nerve cells, neuronal loss, and glial activation (Geronzi et al., 2018). Approximately 26 antiepileptic drugs (AEDs) have been developed that provide effective control of the condition. However, nearly one-third of patients remain resistant to available AEDs and continue to suffer from the disorder (Tang et al., 2017). Furthermore, the narrow therapeutic range of AEDs can result in adverse effects, including affective disorders, cognitive impairment, hepatotoxicity, and recurrent seizures (Baum and Kwan, 2012). Therefore, there is a pressing need for the development of new AEDs that exhibit fewer side effects, enhanced efficacy, and improved safety profiles. Research has demonstrated that oxidative stress plays a role in the pathological processes underlying seizures, exacerbating abnormal neuronal firing and leading to excitotoxicity and oxidative neuronal damage (Tome et al., 2010). Additionally, the brain contains high levels of oxidizable lipids and metals, while possessing limited antioxidant mechanisms, which contributes to its heightened vulnerability to oxidative stress (Bellissimo et al., 2001; Freitas, 2009). Consequently, antioxidants may represent a promising approach to protect neurons from oxidative stress and mitigate epileptogenesis.

Natural antioxidants derived from plants have garnered increasing attention in recent years. CU has been demonstrated to play a beneficial role in epilepsy due to its antioxidant properties, which can alleviate the severity of seizures and reduce oxidative stress in the brain (Jiang et al., 2015; Huang et al., 2020). A randomized, double-blind controlled clinical trial revealed that patients receiving long-term treatment with nanocellular CU experienced a significant reduction in seizure frequency (Erfani et al., 2022). The ability of CU to reduce free radicals facilitates the inhibition of lipoxygenase (LOX), cyclooxygenase (COX), oxidized purinergic alcohols, and nitric oxide synthase (NOS) activities, thereby diminishing the expression of free radicals in various pathophysiological conditions. CU mitigates oxidative stress by forming dimers of dihydrofuran structure with free radicals (Oelkrug et al., 2014). Pentetrazole (PTZ) ignition is widely acknowledged as an animal model for studying epileptogenesis and evaluating antiepileptic drugs, as it lowers the threshold for epileptic seizures by repeatedly stimulating the brain chemically or electrically, ultimately resulting in seizures (Morimoto et al., 2004). Research has shown that pretreatment with CU alleviates seizures, oxidative stress, and cognitive deficits in PTZ-induced ignition in rats (Alam et al., 2023). These findings indicate that CU (300 mg/kg) significantly increased the latency of myoclonic jerks, clonic seizures, and generalized tonic-clonic seizures, improved seizure scores, and reduced the frequency of myoclonic jerks. Furthermore, CU pretreatment reversed the increase in brain malondialdehyde (MDA) levels and the decrease in GSH levels induced by PTZ in a dose-dependent manner (Mehla et al., 2010). Significant reductions in ROS generation, lipid peroxidation, and protein carbonyls were observed in PTZ animals supplemented with CU (Kaur et al., 2014). Similarly, another study demonstrated that CU inhibited the progression of PTZ ignition in mice in a dose-dependent manner by improving the levels of MDA and glutathione (Agarwal et al., 2011). The study reported that CU reduced ROS levels in SH-SY5Y cells (Drion et al., 2018), with the antioxidant effect potentially mediated by the induction of heme oxygenase (Hmox-1), an enzyme that responds to prevent free radical damage and cell death (Gozzelino et al., 2010). CU significantly ameliorated cognitive dysfunction and oxidative damage to the hippocampus and striatum in a persistent epileptic state (SE) model induced by lithium-prusside (Li-Pc). This study also found that pretreatment with CU significantly and dose-dependently attenuated the destruction of antioxidant enzymes in the hippocampus and striatum induced by Li-Pc (Ahmad, 2013). Interestingly, CU has been shown to reduce the expression of TNF-α, IL-10, and TLR4 proteins, as well as MDA levels. This may contribute to its protective effect against febrile convulsions in young mice through its anti-inflammatory and antioxidant properties, along with the downregulation of TLR4 (Atabaki et al., 2020). CU has been shown to ameliorate elevated nitric oxide levels, reduce glutathione levels, and restore catalase activity induced by pilocarpine, while also normalizing Na+, K+-ATPase activity in the hippocampus to control levels (Ezz et al., 2011). This indicates that CU is effective in reducing oxidative stress, and excitability, and inhibiting seizure induction in individuals with epilepsy. Furthermore, dietary intake of CU has been found to inhibit the onset and progression of FeCl_3_-induced seizures, likely due to curcumin’s significant activation of Na^+^, K^+^-ATPase activity and its ability to inhibit lipid and cellular protein damage (Jyoti et al., 2009). Notably, when combined with classical antiepileptic drugs such as valproic acid, phenytoin, phenobarbital, or carbamazepine, CU has been shown to reduce the required doses of these medications without compromising their antiepileptic effects (Reeta et al., 2011). Reeta KH et al. induced epileptic seizures in male rats by electric shock, and injected therapeutic and sub therapeutic doses of phenytoin, phenobarbital, and carbamazepine (AED) before the seizure, or administered CU together with sub-therapeutic doses of AED (Reeta et al., 2011). The results showed that therapeutic doses of AED could completely prevent epileptic seizures. However, sub-therapeutic doses of AED did not completely prevent epileptic seizures. The simultaneous of CU enhanced the protective percentage of AED in sub-therapeutic dose against epileptic seizures and prevented learning and memory impairments caused by seizures, while no such improvement was observed in the group using AED at lower therapeutic doses alone. In addition, the combined of CU would not cause the significant changes in AED serum levels. These findings suggest that CU, as an adjunct to antiepileptic drugs, holds considerable potential in the management of epilepsy, particularly in enhancing efficacy while minimizing dosage and side effects.

## Subarachnoid hemorrhage

Subarachnoid hemorrhage (SAH) is a serious neurological condition characterized by high morbidity and mortality rates, making it the third most common subtype of stroke (Feigin et al., 2009; Sercombe et al., 2002). Approximately 85% of SAH cases result from ruptured intracranial aneurysms (van Gijn et al., 2007). Although the clinical manifestations in SAH patients can vary, the most prevalent symptom is the sudden onset of a thunderclap headache, which is notably painful, unexpected, and severe from the onset of the attack (Perry et al., 2013). Additional conditions may include signs of meningeal irritation, transient or prolonged coma, and focal neurological deficits such as cranial nerve palsy and hemiparesis (Claassen and Park, 2022). Despite significant advances in the understanding of SAH, the prognosis for affected patients remains poor and unsatisfactory. The mechanisms contributing to this unfavorable prognosis are complex and multifactorial, encompassing early brain injury (Pluta, 2005), cerebral vasospasm (Pennings et al., 2004), oxidative stress (Gaetani et al., 1998), inflammation (Fassbender et al., 2001), and diffuse cortical depolarization (Dreier et al., 2009). Research indicates that oxidative stress and lipid peroxidation in cerebrospinal fluid and serum increase in humans 3 days post-SAH (Kamezaki et al., 2002). This phenomenon may result from the oxidation of free extracellular hemoglobin to methemoglobin (MetHb) and its subsequent degradation to heme. Concurrently, free heme catalyzes the production of ROS, leading to oxidative stress that mediates proteolysis, lipid peroxidation, and DNA damage, ultimately resulting in cellular damage and death (Wagner et al., 2003). The inflammatory response induced by oxidative stress may play a crucial role in the pathogenesis of cerebral vasospasm (CVS) following subarachnoid hemorrhage (SAH) (Dumont et al., 2003). In patients with SAH, glutamate levels in cerebrospinal fluid (CSF) were significantly elevated, accompanied by a marked reduction in the expression of glutamate transporter protein-1 (EAAT-2, also known as GLT-1) (Wu et al., 2011). The neurotoxic effects of excessive glutamate include the overactivation of both ionotropic and metabotropic glutamate receptors, which leads to a substantial influx of Ca^2+^ into cells, resulting in apoptosis and necrosis (Lai et al., 2014). Furthermore, oxidative stress contributes to neuronal damage and mediates neuroinflammation, blood-brain barrier (BBB) disruption, and the production of spasminogen following SAH (Ayer and Zhang, 2008). Therefore, therapeutic strategies aimed at mitigating oxidative stress may prove beneficial for patients suffering from SAH.

CU has demonstrated multiple significant neuroprotective effects, particularly in conditions such as ischemic stroke, traumatic brain injury, and intracranial hemorrhage (Dohare et al., 2008; Cole et al., 2007; Thiyagarajan and Sharma, 2004). Notably, several studies indicate that CU may provide a protective effect against cerebral ischemia and cardiovascular spasms following subarachnoid hemorrhage (SAH) through its antioxidative properties (Ghoneim et al., 2002; Wang et al., 2005). In a study involving SAH model rats, oral administration of CU (10 mg/kg) over the course of 1 week resulted in a significant reduction in glutamate and GLT-1 expression, with malondialdehyde (MDA) levels in the hippocampus and cortical regions decreasing by 18% and 29%, respectively. Concurrently, the activities of antioxidant enzymes, including superoxide dismutase (SOD), catalase, glutathione reductase, and lactate dehydrogenase (LDH), were found to increase following CU treatment (Zhang et al., 2016). In another experiment utilizing a double hemorrhage model, SAH rats received an intraperitoneal injection of 20 mg/kg CU for 6 days, leading to improved mortality rates, reduced basal artery wall thickness, decreased neuronal degeneration, and enhanced system scores. These improvements may be associated with the observed reductions in glutamate and MDA levels, alongside increases in SOD and catalase activities within the hippocampus and cortex post-treatment (Kuo et al., 2011). These findings further support the efficacy of multiple CU treatments in counteracting glutamate neurotoxicity and oxidative stress, thereby improving mortality rates. Additionally, another study confirmed that CU significantly inhibited the overexpression of monocyte chemoattractant protein-1 (MCP-1) and tumor necrosis factor-alpha (TNF-α), reduced lipid peroxidation, and restored MDA levels, which collectively ameliorated neurological deficits, alleviated cerebral vasospasm, and significantly decreased mortality in SAH (Cai et al., 2017). Oxyhemoglobin (OxyHb), the primary component of blood, has been reported as a major contributor to cerebral vasospasm and neurological dysfunction in SAH (Luo et al., 2011). The mechanism may involve OxyHb clearing nitric oxide (NO), which leads to vascular spasm (Schwartz et al., 2000). This process activates the Rho/Rho kinase pathway and protein kinase C, resulting in cerebral vasoconstriction (Wickman et al., 2003). Additionally, OxyHb can be oxidized to methemoglobin (MetHb), leading to the production of ROS (Ayer and Zhang, 2008). In a study, cortical neurons were exposed to 10 μM OxyHb for 24 h in the presence of CU. The results indicated that both low and high doses of CU significantly reduced levels of ROS, MDA, tumor necrosis factor-alpha (TNF-α), interleukin-1 beta (IL-1β), interleukin-6 (IL-6), and the Bax/Bcl-2 ratio, while enhancing the activity of SOD and glutathione peroxidase (GSH-Px), thereby increasing cell viability. This suggests that CU attenuates OxyHb-induced oxidative stress and inflammation in neurons, ultimately suppressing apoptosis (Li et al., 2016). Cerebral vasospasm is a major cause of death and disability following subarachnoid hemorrhage (SAH), and it is promoted by oxidative stress (McGirt et al., 2002). A separate study found that CU (150 or 300 mg/kg) prevented cerebral vasospasm and limited secondary cerebral infarction after SAH in mice (Wakade et al., 2009). This effect may be attributed to the significant attenuation of inflammatory gene expression and lipid peroxidation in the cerebral cortex and middle artery.

## Glioblastoma

Brain tumors are prevalent malignant neoplasms of the central nervous system, categorized into gliomas (including astrocytomas, ependymomas, and oligodendrogliomas) and non-gliomas (such as meningiomas and medulloblastomas) (Finch et al., 2021). These tumors are classified into four grades based on histological features. Grade I tumors exhibit moderate proliferative capacity and are typically amenable to surgical intervention. Grade II tumors demonstrate a significant propensity for infiltration and recurrence following treatment. Grade III tumors are characterized by larger atypical nuclear fission. In contrast, Grade IV tumors display vascular proliferation and necrosis, rendering them the most aggressive among brain tumors (Komori et al., 2018; Huttner, 2012). Notably, glioblastoma (GBM, classified as WHO Grade IV) comprises approximately 75%–80% of all brain malignancies and is recognized as the most common and invasive primary malignant brain tumor in adults (Agrawal et al., 2023). Numerous studies have indicated that alterations in redox balance are significant factors in the pathogenesis of GBM (Jain, 2018; Rezaei et al., 2022; Korfi et al., 2021). Elevated intracellular levels of oxidants, such as MDA and ROS, can disrupt the equilibrium between oxidants and antioxidants within cells, leading to increased oxidative stress (Janssen et al., 2000). Research has demonstrated that excessive ROS can ultimately result in both single-stranded and double-stranded DNA damage, impairing cell proliferation and intercellular adhesion, which may contribute to genomic instability and cell death (Sehitogullari et al., 2014). Elevated levels of ROS in cancer cells promote cell proliferation and tumor invasion. Currently, a variety of clinical options are available for the treatment of glioblastoma multiforme (GBM), including surgical resection, radiotherapy, and chemotherapy. The standard treatment regimen for patients with GBM involves maximal surgical resection followed by 6 weeks of radiotherapy and adjuvant chemotherapy with temozolomide (TMZ) (Stupp et al., 2009). Significant progress has been made in both diagnostics and therapeutics. Unfortunately, GBM remains largely resistant to treatment and is associated with a poor prognosis, with an average survival of 15 months after diagnosis and less than 10% of patients surviving 5 years post-diagnosis (Ostrom et al., 2015). Phytochemicals exhibit a wide variety of biological activities, target multiple pathways, and possess relatively low toxicity. Consequently, the incorporation of phytochemicals into current GBM therapy research has garnered widespread attention.

CU is a potential therapeutic agent for GBM that regulates various cellular processes, including GBM cell proliferation, apoptosis, cell cycle stagnation, autophagy, and the migratory capabilities of tumor cells (Shahcheraghi et al., 2019). Previous studies have demonstrated that CU can inhibit ROS-induced tumor formation and protect normal tissues from ROS-mediated DNA damage (Wilken et al., 2011; Daverey and Agrawal, 2016). CU has been shown to inhibit the proliferation and migration of glioblastoma cell lines, including U138MG, U87, U373, C6, U251, U87GB, and T98G, in a dose-dependent and time-dependent manner, while also inducing cell apoptosis, as further confirmed *in vivo* (Wilken et al., 2011; Zanotto-Filho et al., 2012). Glioblastoma stem cells (GSCs) are recognized as a primary contributor to tumor formation, drug resistance, and recurrence; CU has been found to inhibit the viability of GSCs (Gersey et al., 2017). Notably, subtoxic levels of 2.5 μM CU significantly reduce the proliferation, sphere formation ability, and colony formation potential of GSCs. This effect may be attributed to curcumin’s ability to induce ROS, promote activation of the MAPK pathway, and downregulate STAT3 activity and IAP family members (Shehzad and Lee, 2013). Furthermore, as a photosensitive compound, CU possesses a broad absorption peak (300–500 nm) that overlaps with blue light emission. It has also been shown to exhibit photodynamic effects by inducing ROS-mediated apoptosis in the presence of blue light (Leite et al., 2014). Various tumor cells exhibited significant sensitivity to the inhibitory effects of CU photodynamic therapy (PDT) activated by blue LED light (Xie et al., 2022). The photodynamic activation of CU (10 µM) in the presence of blue light resulted in a decrease in matrix metalloproteinases 2 (MMP2) and 9 (MMP9), as well as NF-κB and Nrf2. This cascade led to the activation of ROS-dependent apoptotic pathways in T98G cells, ultimately inducing oxidative stress and cell death (Alkahtani et al., 2023). These findings demonstrate that blue light application enhances the therapeutic efficacy of CU in glioblastoma treatment. Temozolomide (TMZ), a DNA alkylating agent, is commonly used to GBM in clinic. However, therapeutic effect of TMZ is still limited due to the frequent resistance in GBM. Research has found that CU may enhance the therapeutic response of U87MG glioblastoma with TMZ by enhancing cell apoptosis. Moreover, the combination of CU and TMZ has a synergistic effect on the production of reactive oxygen species (ROS) (Yin et al., 2014). However, CU is considered a poor candidate drug due to its low bioavailability, rapid clearance, and metabolism. Most research has focused on developing curcumin analogs and derivatives through chemical synthesis and structural modification to improve therapeutic efficacy, bioavailability, and selectivity. FLDP-5 and FLDP-8, curcumin analogs featuring a piperidone structure, can induce LN-18 cell death in a concentration-dependent manner (IC50: FLDP-5 2.5 µM; FLDP-8 4 μM; CU 31 µM) and inhibit cell migration and invasion (Razali et al., 2022). The involvement of oxidative stress in cell death induced by these analogs was confirmed by a significant increase in intracellular O_2_^−^ and H_2_O_2_ levels, as well as DNA damage observed after 2 and 6 h of exposure. When U87 MG cells were exposed to demethoxycurcumin (DMC) at inhibitory concentrations (0–50 μg/mL), a corresponding increase in ROS production and apoptosis was noted (Kumar et al., 2018). The proposed mechanism suggests that DMC inhibits mitochondrial manganese superoxide dismutase (MnSOD), leading to the production of O_2_^−^, which in turn regulates the Akt/NF-κB signaling pathway associated with cell apoptosis. Notably, the therapeutic potential of CU for GBM has been enhanced through modifications in its dosage form (Gallien et al., 2021; Ghoreyshi et al., 2023). For example, a biodegradable PCL and Mpeg-PCL polymer can encapsulate CU, improving its solubility and absorption both *in vitro* and *in vivo* (Marslin et al., 2017). Although CU is typically regarded as an antioxidant, it can also promote ROS production in cancer cells, thereby inhibiting cell growth. This indicates that CU functions as a modulator of oxidative stress.

## Conclusion

In recent decades, numerous *in vivo* and *in vitro* studies have demonstrated that curcumin, a natural antioxidant, holds significant potential in the prevention, treatment, and adjunctive therapy of neurological diseases. This natural compound has been shown to positively impact conditions such as depression, Alzheimer’s disease, Parkinson’s disease, epilepsy, subarachnoid hemorrhage, glioblastoma, and other neurological disorders through the antioxidant stress mechanism. Evidence indicates that curcumin can effectively alleviate the symptoms of these neurological diseases and moderately delay their progression by combating oxidative stress. Furthermore, CU is regarded as a safe compound with no serious side effects, which favorable lipophilicity enhances the ability to penetrate the blood-brain barrier, thereby exerting pharmacological effects. However, this property also presents challenges associated with poor oral absorption and bioavailability. Preclinical and clinical studies have revealed that CU is unstable under physiological conditions and exhibits suboptimal pharmacokinetic properties, which impede the therapeutic application in clinical settings. To address the issues of poor bioavailability and overall efficacy, researchers have sought to develop novel analogs of CU through structural modifications. A variety of advanced nanoformulations with CU have been developed to enhance bioavailability and targeting (Ullah et al., 2024). However, the therapeutic utility of these nanocarriers remains largely in the preclinical exploration phase. Despite the diverse therapeutic applications of CU, clinical research in neurological diseases is still insufficient and requires further high-quality studies to firmly establish its clinical efficacy. We believe that future research should focus on an in-depth exploration of the mechanisms and signaling pathways associated with CU in neurological diseases. Importantly, optimizing the administration methods and discovering new drug delivery systems are essential for improving bioavailability. Furthermore, clinical trials of CU should be widely promoted to validate the efficacy and safety, thereby providing a more practical reference for the application in neurological diseases.
